# Characterization of local invariances in the ascending ferret auditory system

**DOI:** 10.1186/1471-2202-15-S1-P170

**Published:** 2014-07-21

**Authors:** Jean F Lienard, Stephen V David, Alexander G Dimitrov

**Affiliations:** 1 Department of Mathematics, Washington State University, Vancouver, WA, USA; 2 Oregon Health and Science University, Portland, OR, USA

## 

Local probabilistic invariances, defined by the range of physical transformations that can be applied to a sensory stimulus without changing the corresponding neural response [1], are largely unstudied in auditory cortex. We propose to assess these invariances using existing and new experimental neurophysiological data recorded from multiple stages of the ferret auditory processing hierarchy.

**Figure 1:**
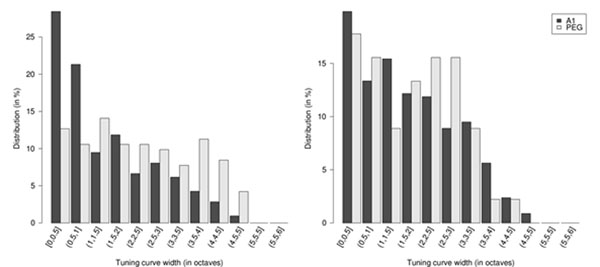
95% confidence interval in frequency for the highest responses in in A1 and PEG. *Left:* with pure tones stimuli. *Right:* with bandpass noise stimuli. The higher values found in PEG compared to A1 demonstrate higher invariance to frequency shifts in PEG than in A1for both stimuli (p < 0.001, one-sided Mann-Withney U test).

We hypothesize that neurons in the auditory pathway will show increasing degrees of local invariance at successively more central stages of the processing hierarchy. To test this hypothesis, we analyzed spiking activity recorded from single neurons in the primary auditory cortex (A1) and in the secondary auditory cortex (PEG) of awake, passively listening animals during presentation of two types of stimuli commonly known to evoke activity in the auditory system. The first set of stimuli was a sequence of isolated pure tones with randomly varying frequency, spanning 5-6 octaves and encompassing the best frequencies of the recorded neurons. The second was a sequence of bandpass noise bursts with varied durations, consisting of 20-30 bursts that logarithmically tile 5-7 octaves, thus achieving a bandwidth each of approximately 0.25 octave. Using these data, we have analyzed local invariance to frequency shifts by estimating the width of the tuning curve for the best responding neurons, and found that the corresponding 95% confidence interval was significantly larger in PEG than in A1 (Figure 1). We have also characterized local invariance to time shifts with the recordings obtained in response to bandpass noise by modeling the temporal jitter of individual neurons as independent processes as in [2,3], indicating as preliminary results that the invariance to time shift is higher in PEG than in A1.
